# The Influence of Cyclic Thermal Shocks at High Temperatures on the Microstructure, Hardness and Thermal Diffusivity of the Rene 41 Alloy

**DOI:** 10.3390/ma17102262

**Published:** 2024-05-10

**Authors:** Elisabeta Roxana Ungureanu Arva, Denis Aurelian Negrea, Andrei Galatanu, Magdalena Galatanu, Sorin Georgian Moga, Daniel-Constantin Anghel, Mihai Branzei, Livia Stoica, Alexandra Ion Jinga, Mircea Ionut Petrescu, Corneliu Munteanu, Marioara Abrudeanu

**Affiliations:** 1 Doctoral School of Material Science and Engineering, National University of Science and Technology Politehnica Bucharest, Splaiul Independenţei Nr. 313, Sector 6, 060042 Bucharest, Romania; elisabetaroxanaungureanu@gmail.com (E.R.U.A.); alexandra.jinga@nuclear.ro (A.I.J.); 2 Nuclear Fuel Plant, Campului Street, Nr. 1, 115400 Mioveni, Romania; 3 Regional Center of Research & Development for Materials, Processes and Innovative Products Dedicated to the Automotive Industry (CRC&D-AUTO), National University of Science and Technology Politehnica Bucharest, University Center Pitesti, Targul din Vale Street, Nr. 1, 110040 Pitesti, Romania; sorin_georgian.moga@upb.ro; 4 National Institute of Materials Physics, Atomistilor 405A, 077125 Magurele, Romania; gala@infim.ro (A.G.); magdalena.galatanu@infim.ro (M.G.); 5 Department of Manufacturing and Industrial Management, National University of Science and Technology Politehnica Bucharest, University Center Pitesti, Targul din Vale Street, Nr. 1, 110040 Pitesti, Romania; dc.anghel@upb.ro; 6 Department of Engineering and Management of Metallic Materials Casting, Faculty of Materials Science and Engineering, National University of Science and Technology Politehnica Bucharest, Splaiul Independenţei Nr. 313, Sector 6, 060042 Bucharest, Romania; mihai.branzei@pub.ro (M.B.); ipetrescu@yahoo.com (M.I.P.); 7 Technical Sciences Academy of Romania, Calea Victoriei, Nr. 118, Sector 1, 010093 Bucharest, Romania; 8 Institute for Nuclear Research, Campului Street, Nr. 1, 115400 Mioveni, Romania; livia.stoica@nuclear.ro; 9 Mecatronics and Robotics Department, Faculty of Mechanical Engineering, Gheorghe Asachi Technical University of Iasi, Bd. Dimitrie Mangeron, Nr. 67, 700050 Iasi, Romania

**Keywords:** superalloy, thermal shock, solar energy, hardness, structural transformations, thermal diffusivity

## Abstract

The precipitation-hardenable nickel-based superalloy Rene 41 exhibits remarkable mechanical characteristics and high corrosion resistance at high temperatures, properties that allow it to be used in high-end applications. This research paper presents findings on the influence of thermal shocks on its microstructure, hardness, and thermal diffusivity at temperatures between 700 and 1000 °C. Solar energy was used for cyclic thermal shock tests. The samples were characterized using microhardness measurements, optical microscopic analysis, scanning electron microscopy coupled with EDS elemental chemical analysis, X-ray diffraction, and flash thermal diffusivity measurements. Structural transformations and the variation of properties were observed with an increase in the number of shocks applied at the same temperature and with temperature variation for the same number of thermal shocks.

## 1. Introduction

Increasing the performance of some engines and installations is frequently related to the operating temperature. In the case of jet engines, achieving an increase in operating performance for heavier payloads, higher speed, and longer ranges is conditioned by high temperatures. In hot plastic deformation in the alloy industry, the durability of the mold and the dimensional accuracy of the parts are determined by the properties of the mold alloy at the processing temperature. In the power generation industry, increased operating temperatures reduce fuel consumption, pollution, and operating costs.

Due to their properties, nickel-based alloys justify their own use in high-end fields, where, exposed to high temperatures, high pressures, and aggressive environments, they must preserve their mechanical characteristics, have dimensional stability, and resist the aggressive action of the environment. The Rene-41 alloy has excellent high-temperature behavior: it is used for static and dynamic applications at temperatures in the range of 650–980 °C, maintaining its mechanical characteristics and surface stability during long exposure [1,2,3].

The properties of Rene-41 are due in large part to the aging heat treatment through which it is produced. The Ni base gives it machinability, excellent mechanical properties, and a convenient cost for the superalloy category. Hardening via alloying the solid solution is achieved by introducing into the solution atoms of different sizes that create elastic, tensile, or compressive stresses in the crystal lattice depending on the relative size difference of the atom to be dissolved compared to the lattice [4].

The alloying elements Al, Ti, Mo, and W improve the mechanical characteristics of the γ solid solution, and Ni and Cr give it excellent high-temperature corrosion properties superior to those of stainless-steel. The structure of the alloy shows a γ austenitic type, a solid solution matrix, with a fcc network. The superior hardening of this alloy is induced by aging, through the secondary precipitation at the grain boundary of the γ solid solution, rich in Cr, Co, and Mo, of the intermetallic compounds that form between Ni, Al, and Ti of the Ni_x_Al_y_ or Ni_x_(Al Ti)_y_ type, called the γ’ phase [5,6,7,8,9,10,11,12,13,14].

The hardening heat treatment consists of a solution quench between 970 °C and 1175 °C, followed by one or more tempering treatments between 600 °C and 815 °C. The γ’ phase is a matrix-coherent precipitate. The carbides formed with the main carburizing elements are characterized by the compositions MC, M6C, M23C6, and M7C3. M23C6 carbide precipitates intergranularly. The secondary phases that form in the structure vary depending on temperature exposure and the speed with which the alloy passes through the solvus temperature. Collins, Quigg, and others [3,4], studying the stability of carbides in Rene-41, showed that the dominant phase at temperatures up to 816 °C and between 1150 °C and 1200 °C is an MC-type carbide, and the M6C phase is dominant between 980 °C and 1150 °C.

During long exposure of Cr-rich alloys to temperatures of around 750 °C, a decomposition process of primary carbides occurs: MC + γ → M23C 6 + γ’ [15].

The amount of M23C6 increases with temperature up to 870 °C and starts to become unstable at 980 °C. The carbides positively influence the behavior of the alloy at high temperatures due to the intercrystalline precipitation that reinforces the grain boundaries.

During heat treatment, recrystallization processes occur along with the formation of double grain boundaries [16,17].

Cyclic thermal requirements with non-uniform heating and cooling, or non-uniform heating and cooling cycles, determine the occurrence of internal stresses. During uneven heating and cooling, voltages of opposite signs appear in the upper layers of the metal and in the core. A number of repeating uneven heating–cooling processes can cause thermal fatigue cracking. The heat transfer properties of an alloy have a decisive influence on the temperature differences in a section and the appearance of internal stresses.

The results presented in this article concern the influence of cyclic thermal shocks on the microstructure, hardness, and thermal diffusivity of the superalloy Rene 41, properties that are essential for this material when using it in applications that require high-temperature operating conditions (e.g., jet engines or applications in the power generation industry).

## 2. Materials and Experimental Technique

The samples used for thermal shock testing have a parallelepipedal shape with a square base with 7 mm side length and a height of 10 mm. The height of the sample is oriented according to the deformation direction of the alloy.

**Figure 1:**
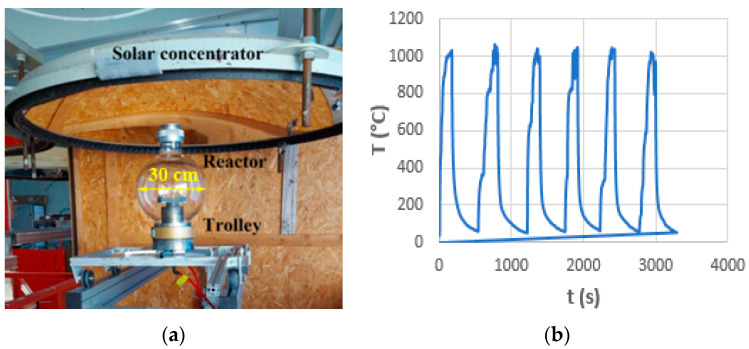
Thermal shock testing with solar energy: (**a**) solar concentrator; (**b**) treatment cycle for 1000 °C, with 6 cycles.

Thermal shock testing was carried out in the solar furnace of the PROMES Laboratory, Font Romeu, Odeillo, France, within the FP7 Specific Program Capacities Project “Study of variation of the mechanical properties of superalloys Inconel 718 and Rene 41 under thermal shock”, TERMOINCORENE P1601300180, SFERA 2016. Cyclic thermal shocks were used in the temperature range of 700–1000 °C, with a constant temperature duration of each cycle of 30s (Figure 1).

The thermal transport properties were investigated from room temperature up to 1050 °C using a Laser Flash Analyzer (Netzsch LFA 457 Microflash). The LFA equipment allows the direct measurement of thermal diffusivity, α, by analyzing the temperature variation of one surface of a sample when a calibrated laser pulse is applied to the other surface. The specific heat of a material, Cp, can be determined via a differential method using a reference sample (in this case, Mo, with the NIST certificate SRM781). The thermal conductivity was calculated as λ = α × ρ × Cp, where ρ is the density of the sample. The samples’ density was measured via Archimedes’ method using a high-resolution balance. The manufacturer for the LFA-457 and DIL-402C is Netzsch GmbH, Selb, Germany.

Microhardness measurements were performed with a FALCON 500 series Micro Vickers device (Vickers, Micro Brinell equipment). For microstructural analysis, samples were prepared via mechanical grinding and attacked with Adler’s reagent. OLYMPUS BX51M bright-field (BF) and dark-field (DF) optical microscope was used at 100×, 200×, and 500× magnification. For this microscope the manufacturer is OLYMPUS, Tokyo, Japan.

Characterization via SEM-EDS was carried out using a HITACHI SU5000 electron microscope equipped with a backscattered electron detector and an energy-dispersive fluorescence spectroscopy module for elemental analysis. For this one, the manufacturer is HITACHI, Tokyo, Japan.

The X-ray diffractograms for the analyzed samples were acquired using a Rigaku Ultima IV diffractometer with a vertical goniometer under the following conditions: Bragg–Brentano mounting, one-dimensional D/teX Ultra detector, and graphite monochromator on the diffracted beam; acceleration voltage of 45 kV and filament current of 40 mA; and a scan range of 2θ [25°–100°], with a step of 0.05° and a scan speed of 2 °/min. For this equipment the manufacturer is Rigaku, Tokyo, Japan. Qualitative phase analysis was performed using PDXL2 integrated software (Rigaku, version 2.8.4.0) and PDF4+ 2023 database (ICDD).

## 3. Experimental Results

### 3.1. The Characterization of the Standard Alloy Rene-41 

The characterization of Rene-41 in the delivery state (standard) was carried out using physical property determinations, HV hardness measurements, morphological characterization via optical and electron microscopy, EDS elemental chemical composition determinations, and XRD qualitative analysis.

**Figure 2:**
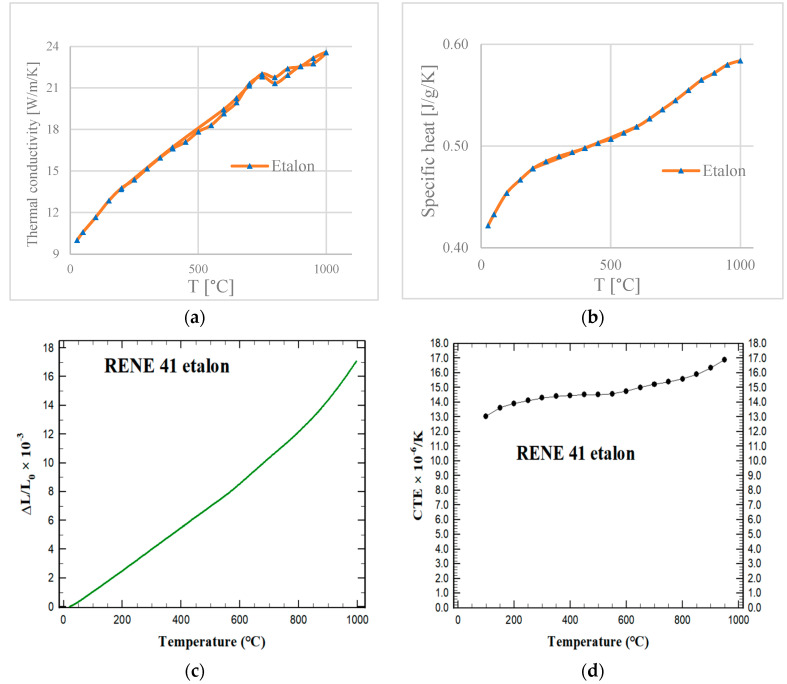
The variation of the physical properties of Rene-41 with temperature up to 1000 °C: (**a**) specific heat, (**b**) thermal conductivity, (**c**) fractional change in length, and (**d**) thermal expansion.

The physical properties of the alloy were characterized by determining the variation in specific heat, thermal conductivity, fractional change in length, and thermal expansion up to 1000 °C (Figure 2).

For thermal diffusivity and specific heat, the measurement was performed at a constant temperature after a thermal equilibrium of 0.1 °C was achieved. Between steps, heating/cooling was induced at 3 °K/min. For thermal expansion, the heating rate was 3 °K/min within the entire temperature interval. The CTE was calculated for selected points, taking the reference temperature to be 20 °C.

The hardness measured along the height of the sample, following a direction parallel to the deformation direction, had an average value of 375 HV.

The determined chemical composition (in the scanned area and at three points on the surface) and the standard chemical composition are shown in Table 1 [18].

**Figure 3:**
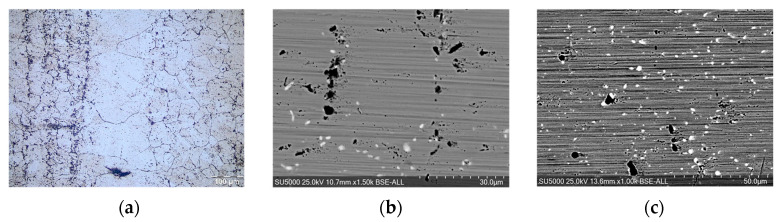
Microstructure of the standard sample in the initial state: (**a**) MO, magnification 100×; (**b**) SEM 1.5k×; (**c**) SEM 1k×.

The microstructure of the alloy in its initial state is a strip structure, typical of plastic deformation, with hard phases oriented along the direction of deformation and a matrix with a polyhedral structure (Figure 3). The bands containing precipitates at the grain boundary have a fine structure, and the band of solid solution γ shows large grains with grain boundaries that are not well defined (Figure 3a). Its precipitates are of two types: large, dark-colored precipitates and fine, light-colored precipitates (Figure 3b,c).

**Figure 4:**
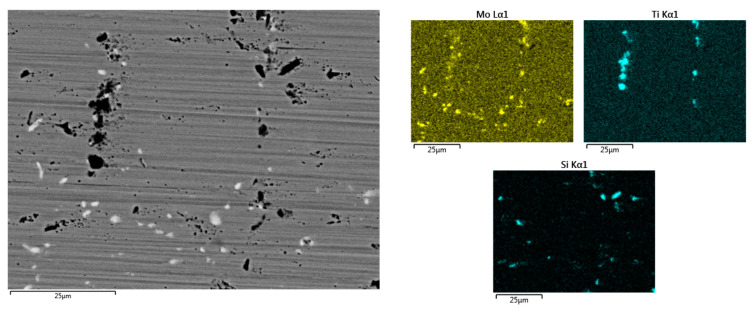
SEM-EDS mapping analysis of Rene-41 standard sample.

The results of EDS analysis along with the element distribution map shows that the dark precipitates are rich in Ti, and the light-colored ones contain a lot of Mo (Figure 4).

Qualitative analysis of the standard sample using X ray-diffraction highlighted two phases: the γ matrix, with space group Fm3m (225) and a FCC structure [19], and the intermetallic compound γ’ (Ni_3_(Al,Ti)) [20], corresponding to PDF card no. 01-085-8671. Mo carbides were not identified.

### 3.2. Morphology and Elemental Chemical Composition of Samples Subjected to Thermal Shocks

The morphologies and chemical element compositions of samples subjected to thermal shocks are presented below.

**Figure 5:**
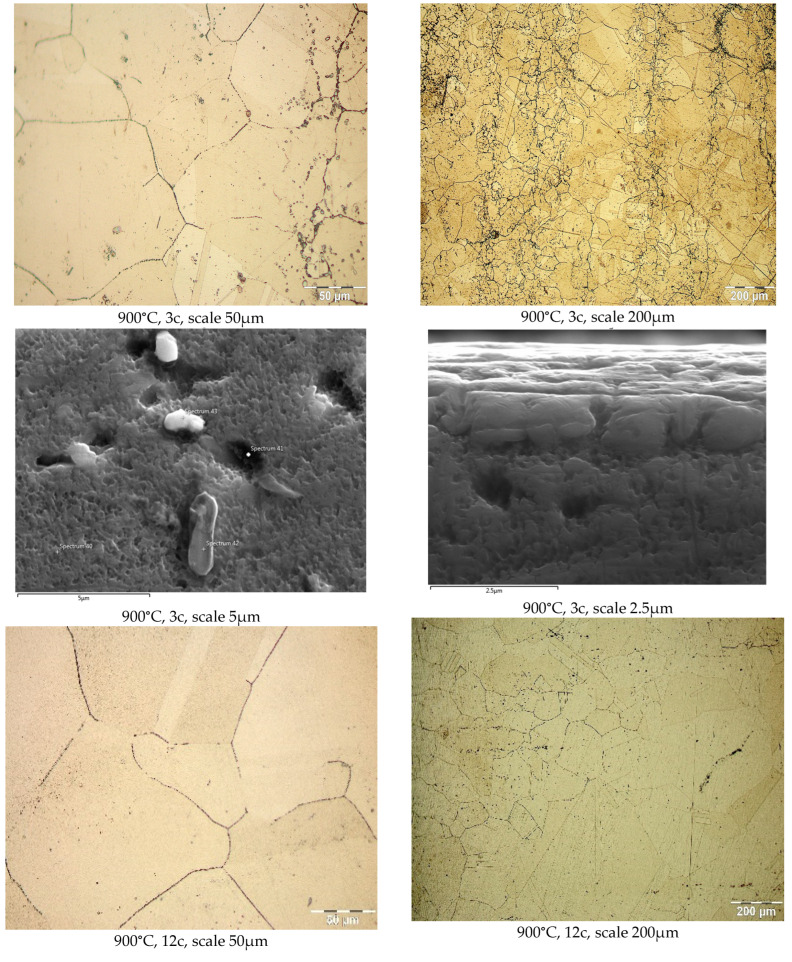

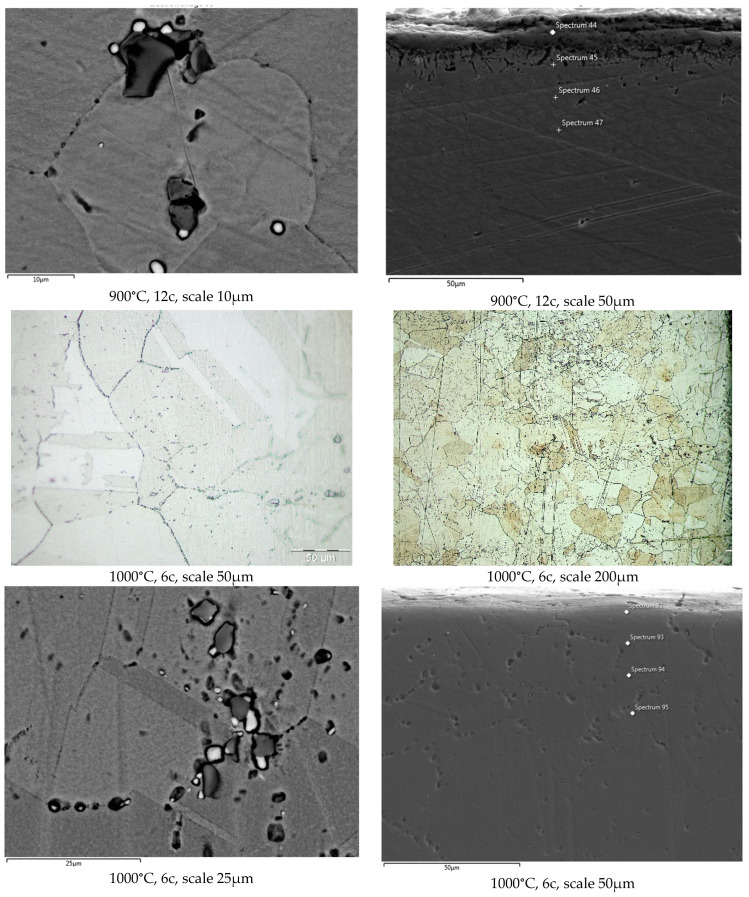

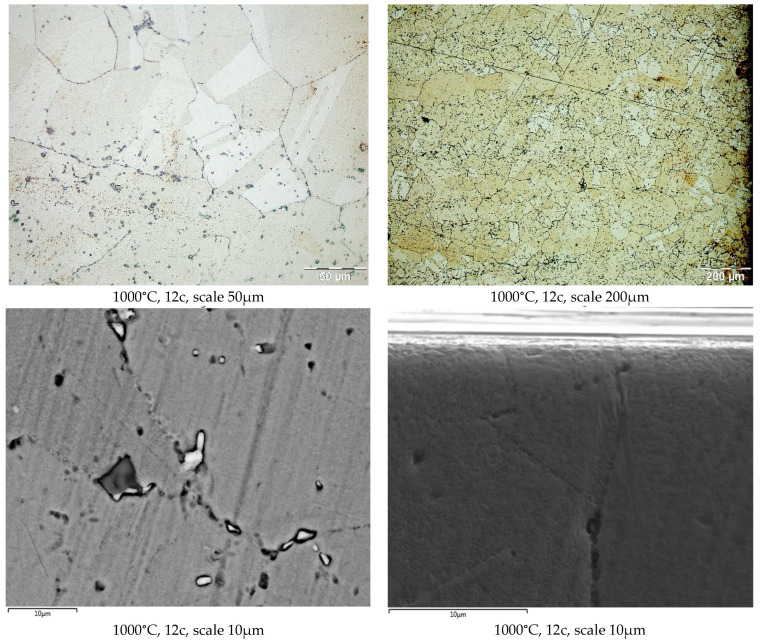
The influence of thermal shocks on the microstructure in the vicinity of the shock surface, where “c” is an abbreviation for the thermal shock cycles.

The influence of temperature and the number of thermal shock cycles on the microstructure in the vicinity of the shock application surface is shown in Figure 5.

**Figure 6:**
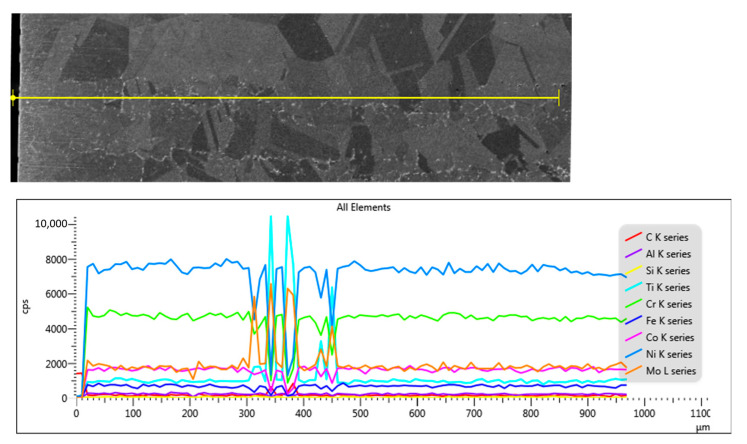
SEM-EDS line-scan analysis of the cross-section for the sample treated at 700 °C with 3c.

The SEM EDS analysis in section shows a location in the same area of the precipitates rich in Ti and Mo (Figure 6).

**Figure 7:**
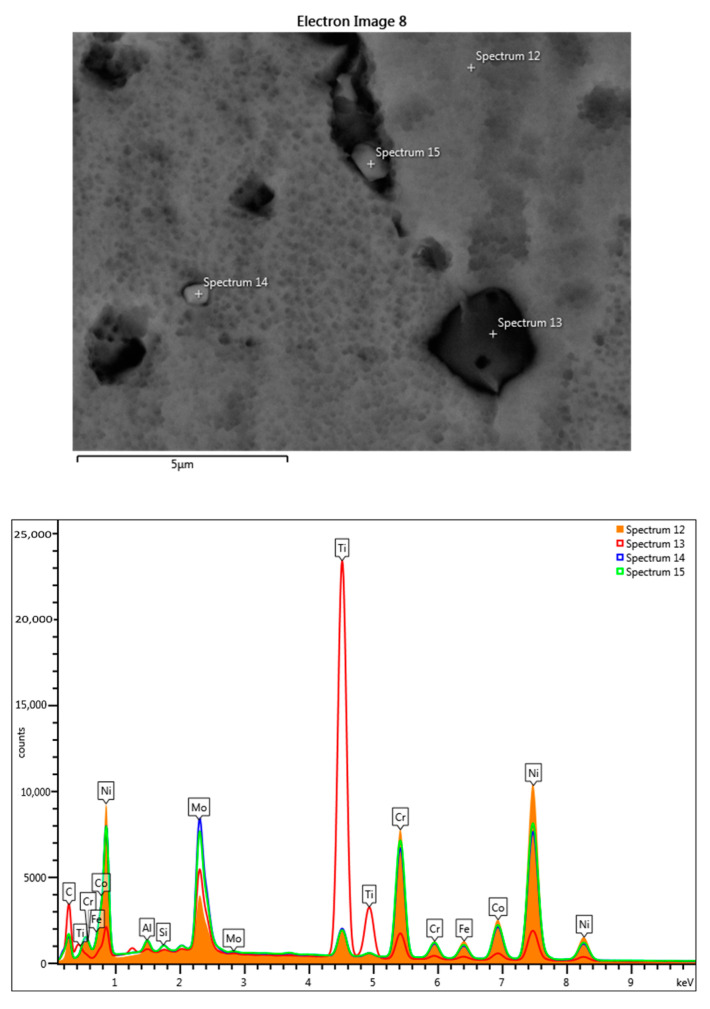
SEM-EDS point-scan analysis and the obtained superimposed spectra for the sample treated at 700 °C for 3c: spectrum 12—matrix, spectrum 13—Ti-rich compounds, and spectra 14 and 15—Mo-rich compounds.

The chemical element analysis of the section scan of the sample treated at 700 °C for 3 cycles confirmed that the precipitates located at the grain boundary mainly contain Ti and Mo, respectively (Figure 7 and Table 2).

**Figure 8:**
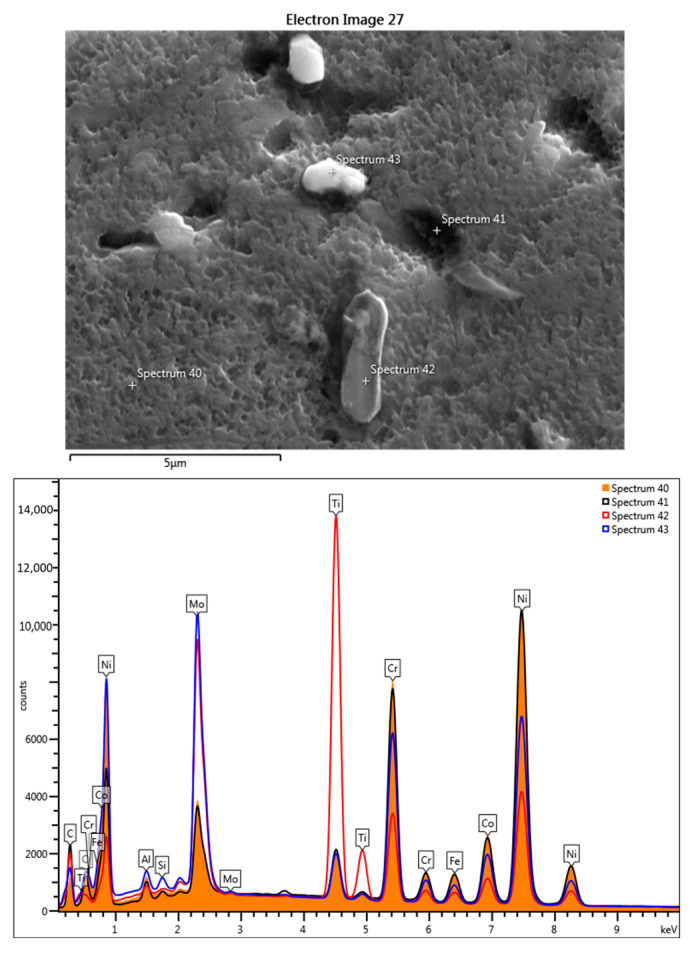
SEM-EDS point-scan analysis and the obtained superimposed spectra for the sample treated at 800 °C for 3c.

**Figure 9:**
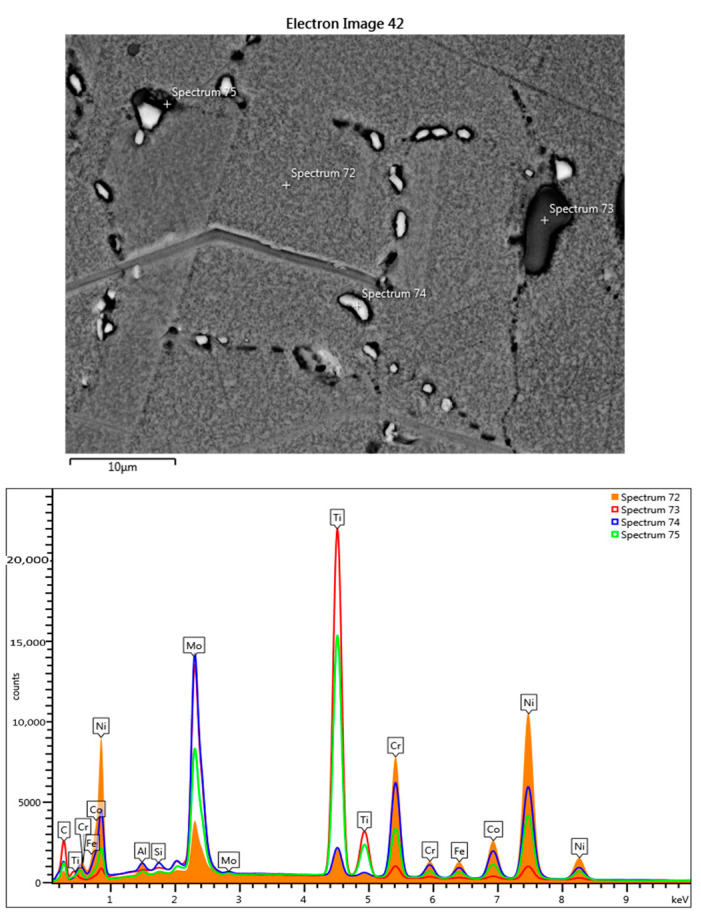
SEM-EDS point-scan analysis, the obtained superimposed spectra, and the obtained elemental composition for the sample treated at 900 °C for 3c.

The three-cycle heat-shocked samples contain the same dark Ti-rich and light Mo-rich phases (Figure 8). Increasing the thermal shock temperature by 100 °C led to of the increase of Mo content in the Ti-rich phases formed at 900 °C (Figure 9, Table 3, spectra 73 and 75); andthe light-colored phases in the grain boundary are carbides of Mo and Cr (Figure 9, Table 3, spectrum 74).

The precipitation of secondary phases for the sample with thermal shocks applied at 900 °C occurred along the initial grain boundaries.

**Figure 10:**
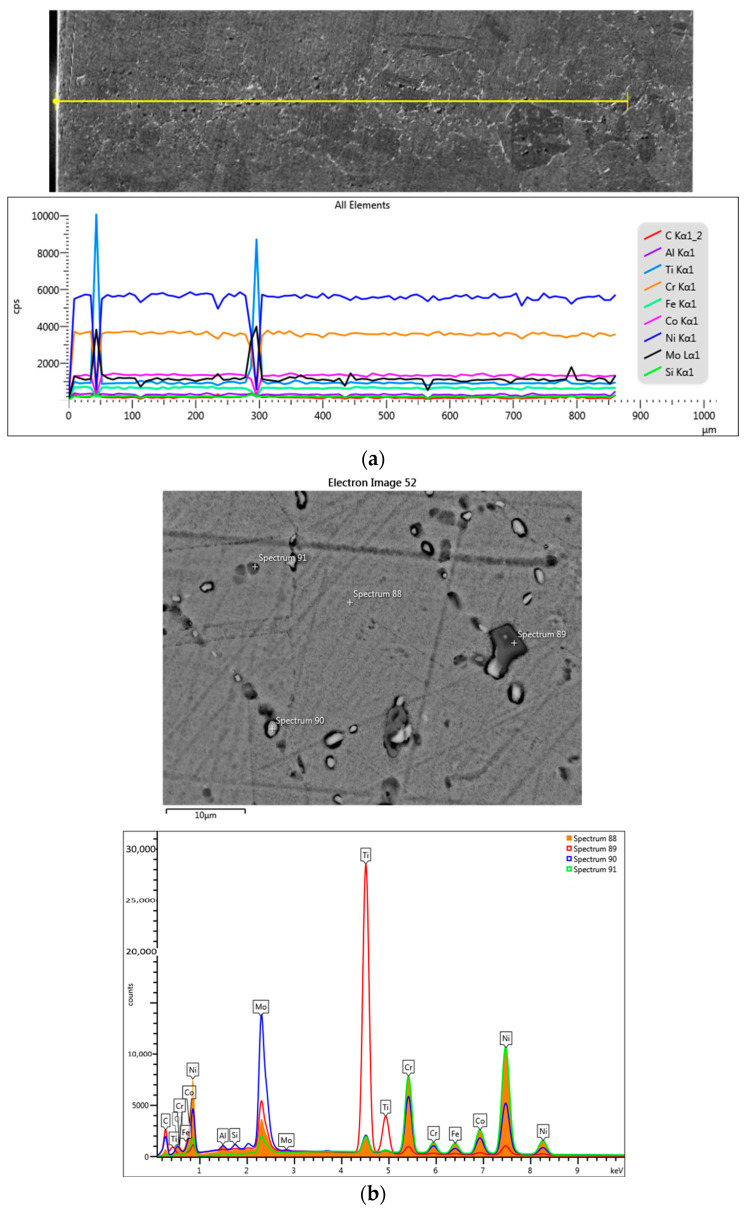
SEM-EDS analysis of the sample treated at 900 °C for 12c: (**a**) line-scan; (**b**) point-scan analysis and the obtained superimposed spectra.

**Figure 11:**
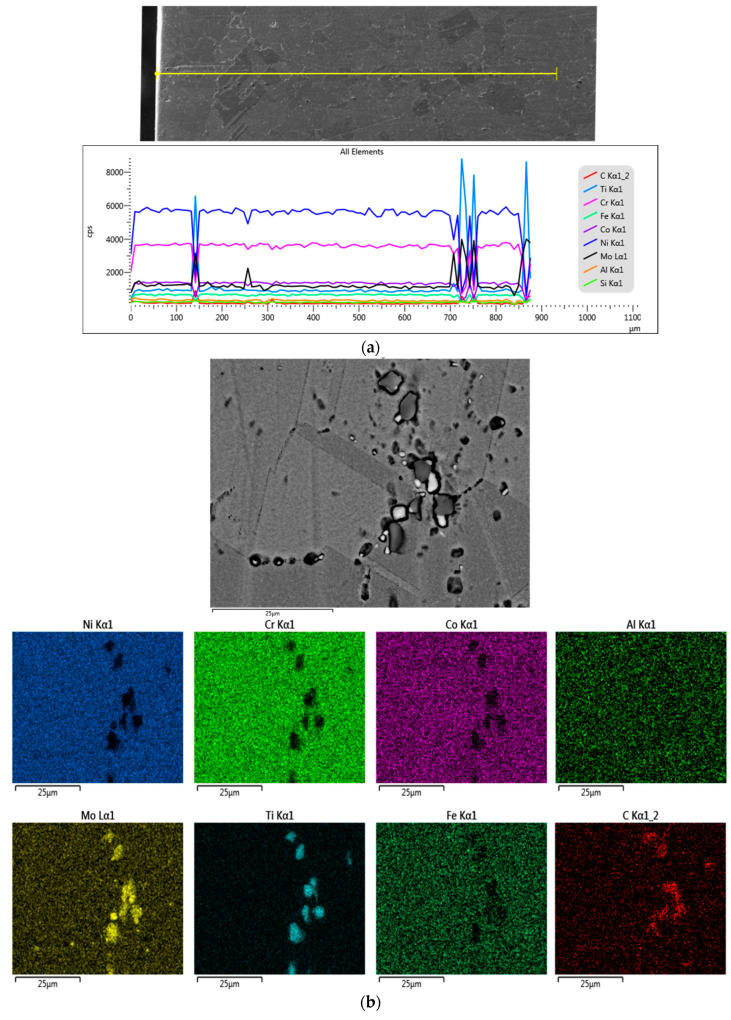

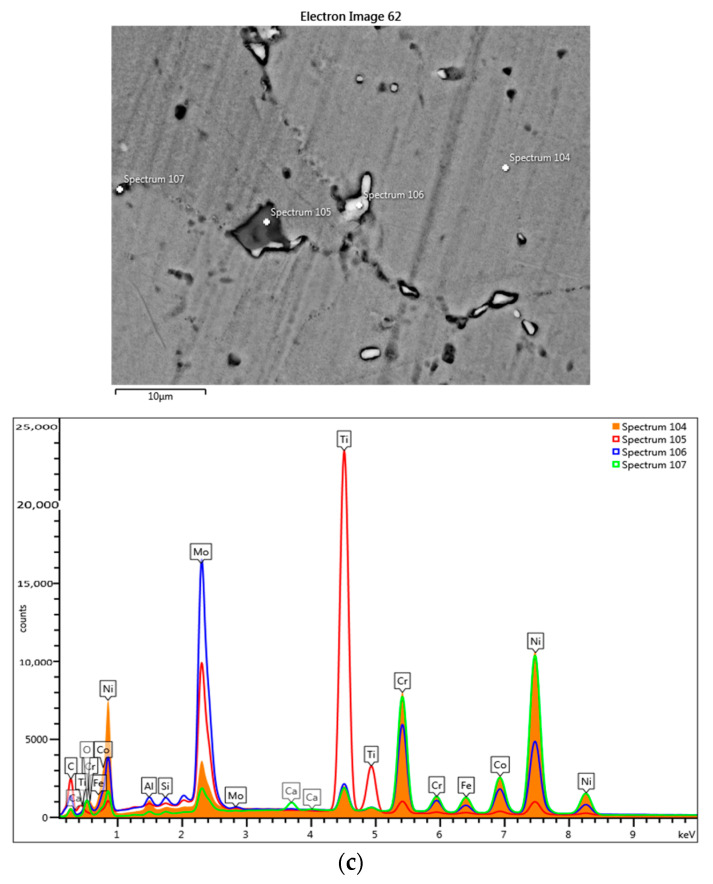
SEM-EDS analysis of the sample treated at 1000 °C for 6 cycles: (**a**) line-scan; (**b**) mapping, and (**c**) point-scan analysis and the obtained superimposed spectra for the sample treated at 1000 °C for 12c.

In the case of the samples treated at 900 °C (Figure 10) and at 1000 °C (Figure 11), the precipitation of the secondary phases in the boundary of the initial grains presented discontinuities, forming a structure with discontinuous, interrupted boundaries, also called a “serrated grain boundary” structure [21]. The formation of a jagged structure causes a decrease in strength by potentiating the development of cracks [22,23].

The online scan of the sample section and the elemental composition map clearly highlight the presence of C, Mo, and Cr in the grain boundary, where there are precipitates in the form of carbides and Mo is present alongside Ti in the large precipitates (Figure 11).

### 3.3. Qualitative Phases Analysis via X-ray Diffraction

**Figure 12:**
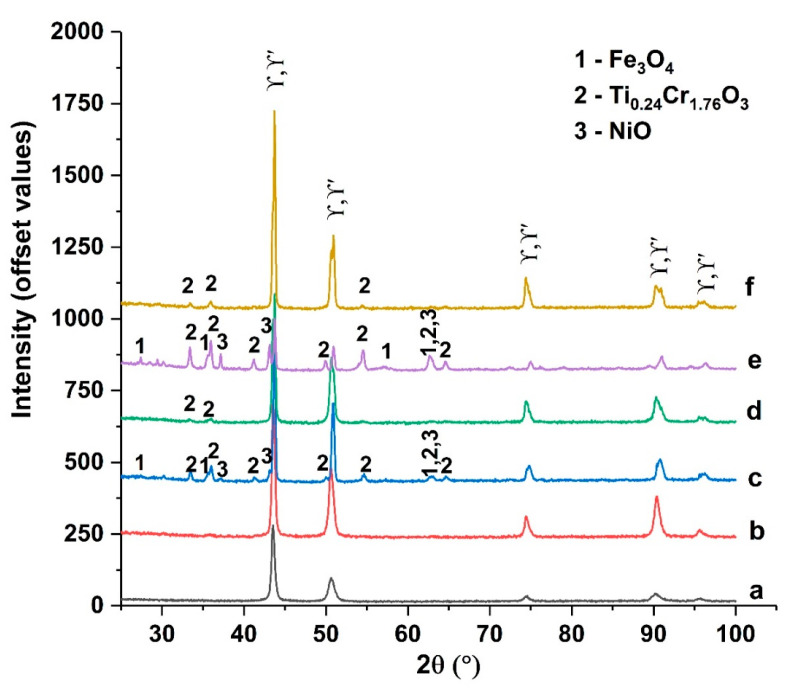
Diffractograms for Rene-41 standard—a—and samples treated with thermal shocks: 700 °C with 3 cycles—b; 700 °C with 9 cycles—c; 800 °C with 3 cycles—d; 800 °C with 9 cycles—e; and 900 °C with 3 cycles—f.

**Figure 13:**
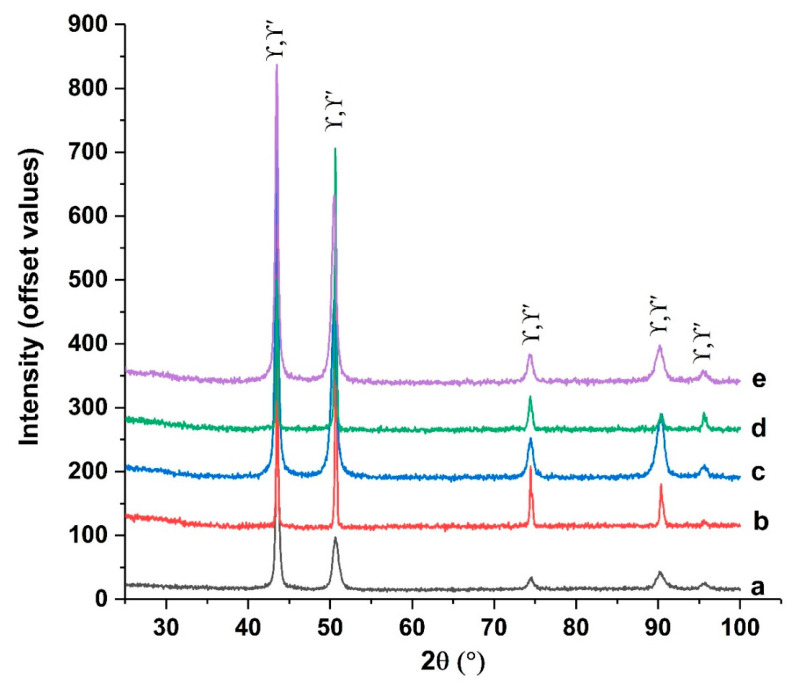
Diffractograms for Rene 41 standard—a—and samples treated using thermal shocks: 900 °C with 6 cycles—b; 900 °C with 12 cycles—c; 1000 °C with 6 cycles—d; and 1000 °C with 12 cycles—e.

The X-ray diffractograms (Figure 12 and Figure 13) of the analyzed samples mostly show diffraction lines associated with a γ matrix; Fm3m space group (225), with a FCC structure [19]; and the intermetallic compound γ’ (Ni3(Al,Ti)) [20], with the PDF card no. 01-085-8671. The main elements that form precipitates (with Ni) in the structure of Rene alloys are Al and Ti [19].

XRD qualitative phase analysis showed the presence of some oxides on the surfaces of the thermal shocked samples:

- For the samples treated at 700 °C with nine cycles (c) and at 800 °C with nine cycles (e): Fe_3_O_4,_ PDF card no. 01-084-2782; Ti_0.24_Cr_1.76_ O_3_, PDF card no. 04-015-9779; and NiO, PDF card no. 04-004-8992.
- For the samples treated at 800 °C with three cycles (d) and at 900 °C with 3 cycles (f): only Ti_0.24_Cr_1.76_ O_3_, PDF card no. 04-015-9779.

Iron (Fe_3_O_4_) and nickel (NiO) oxides or complex chromium and titanium oxides (Ti_0.4_Cr_1.76_O_3_) were identified in the upper layers of the surfaces to which thermal shocks were applied. Heat-shocked structures at lower temperatures retain bands of precipitates from the original structure, which decrease in number as the shock temperature increases and larger precipitates form.

The constituent phases for the samples subjected to thermal shocks at temperatures above the solution temperature (900 °C and 1000 °C) are identical to those present in Rene 41 in its initial state.

### 3.4. Influence of Thermal Shocks on Hardness

Hardness measurements were carried out along the height of the samples, from the surface opposite the shock to the shock application surface.

**Figure 14:**
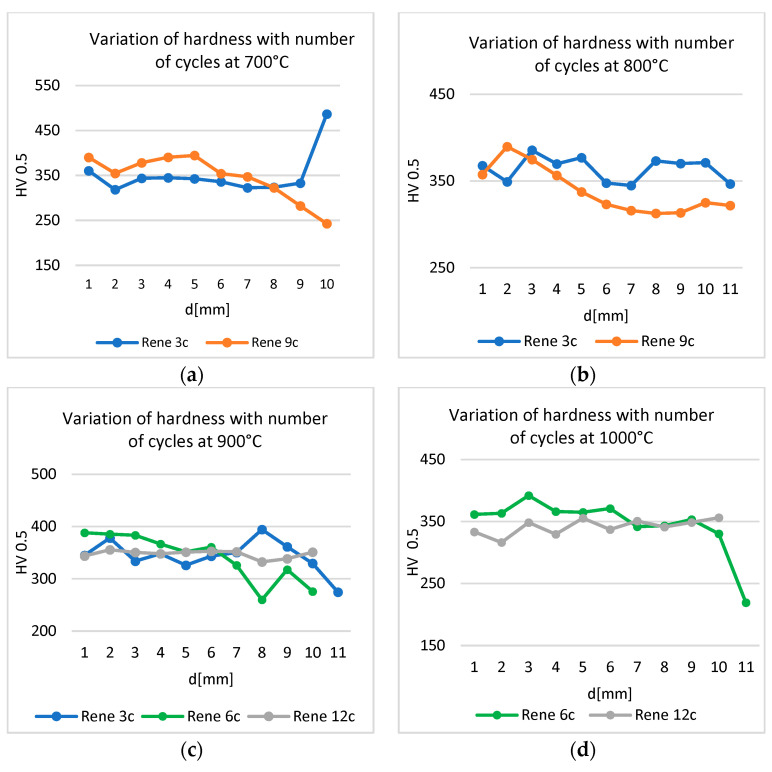
Variation in hardness with the number of thermal cycles applied at (**a**) 700 °C, (**b**) 800 °C, (**c**) 900 °C, and (**d**) 1000 °C.

The hardness of the samples decreased from the opposite surface to the surface on which thermal shocks were applied. For the same temperature, the hardness in the vicinity of the shock application decreases with the increase in the number of cycles (Figure 14). The exception is the sample treated at 1000 °C: after 12 cycles, the hardness stabilized around 350 HV, with a slight increase (Figure 14a).

**Figure 15:**
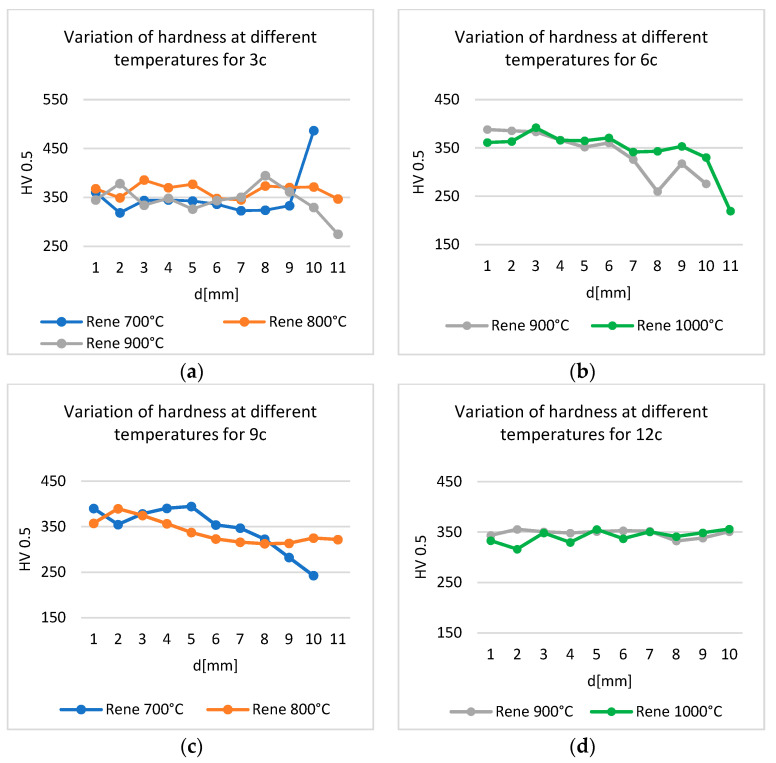
Variation in hardness for different temperatures and the same number of thermal cycles: (**a**) 3 cycles, (**b**) 6 cycles, (**c**) 9 cycles, and (**d**) 12 cycles.

For the same number of cycles, the hardness in the vicinity of the treatment zone decreases with increase in heat shock temperature (Figure 15).

The hardness values, determined along the height of the sample, from the untreated surface to the one to which the shock was applied, depending on the number of thermal shocks applied at the same temperature, register a decrease in the vicinity of the shock surface. The exception is the sample treated at 700 °C with three cycles (Figure 15a), which registered an important increase, explained by the completion of the precipitation process in the immediate vicinity of the shock surface.

### 3.5. The Influence of Thermal Shocks on Thermal Diffusivity

For all the samples, a variation in thermal diffusivity was observed during heating in the range of 20–1000 °C. The values are compared with the diffusivity variation of the reference sample. All values determined after treatment at up to 1000 °C are superior to those for the standard sample.

The vertical gap between the diffusivity values at the beginning of the temperature variation curve of the heat-shocked samples and the curve for the reference sample represents the difference in diffusivity, at ambient temperature, for the samples treated via thermal shock in relation to the untreated reference sample.

**Figure 16:**
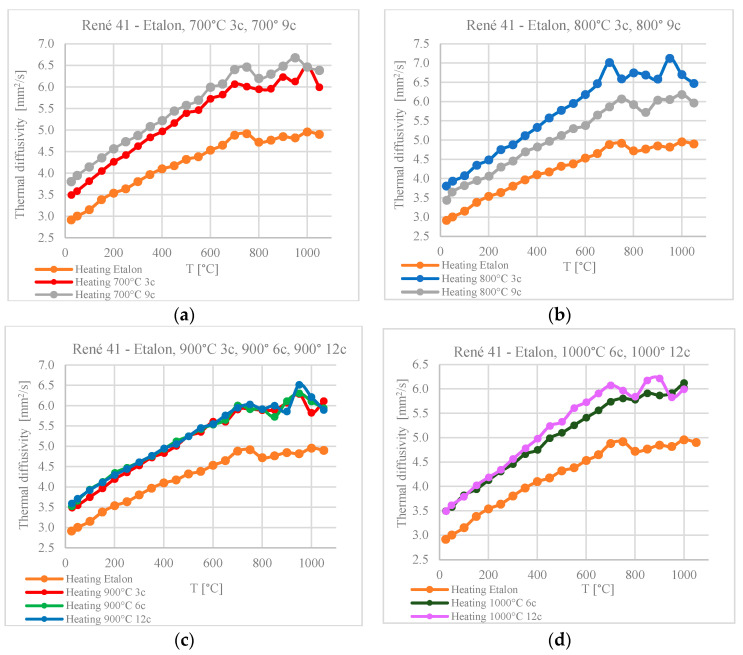
Variation in diffusivity with the number of cycles for the same thermal shock temperature: (**a**) 700 °C, (**b**) 800 °C, (**c**) 900 °C, and (**d**) 1000 °C.

For the same thermal shock application temperature, the thermal diffusivity values measured in the range of 20–1000 °C increase with the number of applied cycles (Figure 16). The exception is the samples treated at 800 °C, for which the diffusivity values of the sample with three thermal shock cycles are superior to those of the sample treated with nine shock cycles.

**Figure 17:**
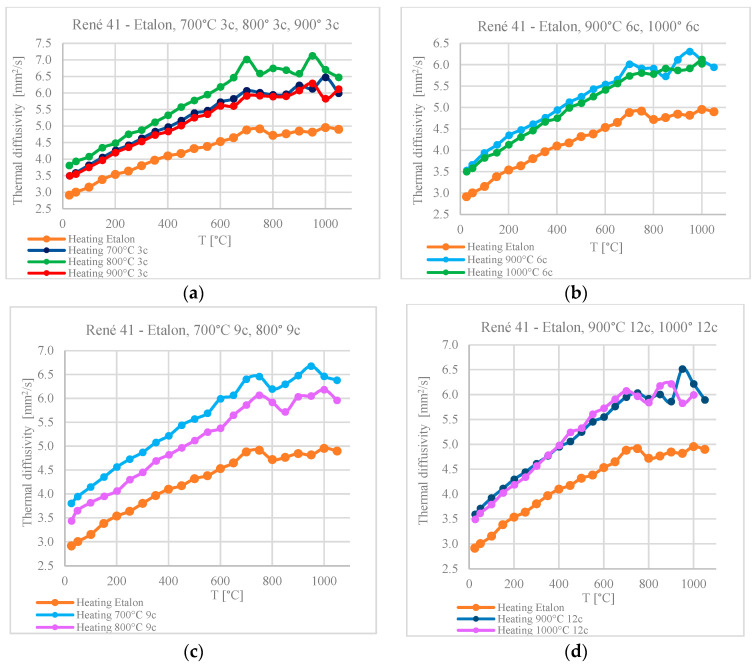
Variation in diffusivity with temperature for the same number of thermal shocks: (**a**) 3 cycles applied at 700°, 800°, and 900 °C; (**b**) 6 cycles at 900° and 1000 °C; (**c**) 9 cycles at 700° and 800 °C; (**d**) 12 cycles at 900° and 1000 °C.

For the same number of thermal shock cycles, the diffusivity decreases with an increasing shock application temperature (Figure 16). The exception is the sample treated in the range of 800 °C with three cycles (Figure 17a). This exception could be associated with the structural transformations that take place in the alloy in the range of 760–800 °C [24,25].

In the analysis of the influence of cyclic thermal shocks on the microstructure and hardness of these alloys, we must take into account the influence of thermal shocks on heat transfer processes. Thermal diffusivity values were determined with temperature variation from ambient temperature to 1000 °C.

For the same shock temperature, the diffusivity values lie on close curves, for which higher values correspond to a higher number of thermal cycles. The exception is the samples treated at 800 °C, which form maxima in the temperature range of recovery, corresponding to solubilization.

The variation in thermal diffusivity with increasing shock temperature, at the same number of applied cycles, follows offset curves for shock application temperatures of 700 °C and 800 °C or superimposed curves for shock temperatures of 900 °C and 1000 °C. Unlike the standard sample, which shows a single maximum in the diffusivity variation with the measurement temperature, in the 600–800 °C temperature range the samples subjected to thermal shocks also show the second maximum value for thermal diffusivity around 900 °C. 

## 4. Discussion of Results

Rene 41, a nickel-based heat-resistant superalloy (HRSA), is recommended for use within the temperature range of 680–980 °C. Its exceptional properties are due to the association of an important number of alloying elements that confer remarkable mechanical characteristics and high resistance to oxidation. Its high content of alloying elements influences its heat transfer properties, which are particularly important for evaluating the usefulness of an alloy for high-temperature applications.

The phases present in the microstructure of the alloy are the highly alloyed solid solution matrix ɣ; Ni-Ti-Al compounds; ɣ’, precipitated after the structural hardening treatment; and the Cr, Co, and Mo carbides precipitated in the grain boundary [9,10,11,12,13,14]. The SEM-EDS analyses highlighted the distribution of the ɣ’ phase in the form of small particles in the grain boundary and in the form of large intragranular particles. The precipitation of secondary phases at high temperatures occurred partially along the initial grain boundaries, forming a structure with serrated grain boundaries.

According to the studies carried out by Glotka et al. [8] at high Mo content, carbides can form films that worsen mechanical characteristics. The SEM-EDS analyses carried out highlighted the presence of molybdenum both at the grain boundary in the form of rounded particles (without film formation) but also in the area of large intragranular particles containing titanium. In the analyzed samples, the carburizing elements distributed in the grain boundary take the form of small, rounded particles that ensure boundary stability and creep resistance [17].

Microhardness measurements showed a decrease in hardness in the thermally affected area with the increase in the temperature of the shocks and the number of applied thermal shocks. The exception found for the shock temperature of 700 °C, located in the annealing temperature range [5,6,7,8], can be explained by precipitation in the completion of the annealing process.

Few references were found in the literature regarding the thermal diffusivity of nickel-based superalloys for: determinations regarding the influence of aluminum content on diffusivity through the percentage of the ɣ’ phase, determinations and estimation correspondent to the solid–liquid transition domain [26,27,28,29,30], and a comparison study between these type of alloys in the 20–1200 °C temperature range [31]. This paper reports the variation in the thermal diffusivity of Rene 41 determined by measurements in the range of 25–1000 °C performed for samples previously subjected to cyclic thermal shocks at temperatures between 700 and 1000 °C in comparison to the variation in the sample’s diffusivity in the delivery state. The variation curves determined are similar to the curve developed by Quested et al. [31].

This study provides additional information regarding the influence of temperature on thermal transfer and the behavior of the studied material when subjected to cyclic thermal shocks carried out at high temperatures.

## 5. Conclusions

As novel elements of the present study, we mention the following:

- The properties of the Rene 41 superalloy subjected to cyclic thermal shocks at high temperatures (700–1000 °C) were determined
- The characterization of the evolution of this material’s microstructure with respect to the number of shocks and the application temperature of the cyclic thermal shocks;
- The establishment of the evolution of the microhardness of Rene 41 when subjected to cyclic thermal shocks;
- The determination of the variation in the physical properties (specific heat, thermal conductivity, fractional change in length, thermal expansion, and thermal diffusivity) of Rene-41 in the delivery state in the range of 25–1000 °C;
- The determination of the variation in the thermal diffusivity of Rene 41 when subjected to cyclic thermal shocks under conditions of increasing the shock temperature and the number of shocks applied in proportion to the variation in the diffusivity of the alloy in the delivery state in the range of 25–1000 °C.

The experimental data contribute to the enrichment of the database regarding the behavior of Rene 41 at high temperatures.

## Figures and Tables

**Table 1:** Chemical composition of Rene-41.

	Concentration (wt%)												
Element	Al	Ti	Cr	Mn	Fe	Co	Ni	Mo	Si	S	Cu	B	C
Determined composition	1.62	3.33	19.16	0.19	3.52	10.48	51.01	10	0.69	-	-	-	-
Standard composition	1.4–1.8	3.0–3.3	18–20	<0.1	<5.0	10–12	bal	9.0–10.5	<0.5	<0.015	<0.5	0.003–0.01	0.06–0.12

**Table 2:** Chemical compositions obtained for the 4 points from Figure 7.

Spectrum Label	Spectrum 12 [%wt]	Spectrum 13 [%wt]	Spectrum 14 [%wt]	Spectrum 15 [%wt]
Al	1.98	0.37	1.47	1.53
Si	0.19	0.20	0.59	0.54
Ti	3.39	62.75	3.86	3.49
Cr	19.13	6.17	18.04	18.76
Fe	3.53	0.87	2.89	3.05
Co	10.18	2.35	8.89	9.18
Ni	50.47	11.15	38.32	40.26
Mo	11.13	16.15	25.94	23.20
Total	100.00	100.00	100.00	100.00

**Table 3:** Chemical compositions obtained for the 4 points from Figure 9.

Spectrum Label	Spectrum 72 [%wt]	Spectrum 73 [%wt]	Spectrum 74 [%wt]	Spectrum 75 [%wt]
Al	1.87	0.10	0.92	0.59
Si	0.17	0.14	0.63	0.18
Ti	3.48	56.06	4.12	37.01
Cr	19.13	2.74	16.26	10.21
Fe	3.51	0.32	2.43	1.73
Co	10.40	0.94	7.62	4.57
Ni	50.66	4.56	27.93	21.76
Mo	10.79	35.14	40.09	23.96
Total	100.00	100.00	100.00	100.00

## Data Availability

All data results are presented in the present article.
